# Change of Renal Gallium Uptake Correlated with Change of Inflammation Activity in Renal Pathology in Lupus Nephritis Patients

**DOI:** 10.3390/jcm10204654

**Published:** 2021-10-11

**Authors:** Tsu-Yi Hsieh, Yi-Ching Lin, Wei-Ting Hung, Yi-Ming Chen, Mei-Chin Wen, Hsin-Hua Chen, Wan-Yu Lin, Chia-Wei Hsieh, Ching-Tsai Lin, Kuo-Lung Lai, Kuo-Tung Tang, Chih-Wei Tseng, Wen-Nan Huang, Yi-Hsing Chen, Shih-Chuan Tsai, Yi-Da Wu

**Affiliations:** 1 Division of Allergy, Immunology and Rheumatology, Taichung Veterans General Hospital, Taichung 40705, Taiwan; zuyihsieh@gmail.com (T.-Y.H.); wtinghung@gmail.com (W.-T.H.); blacklark@gmail.com (Y.-M.C.); shc5555@hotmail.com (H.-H.C.); chiaweih@gmail.com (C.-W.H.); chingtsia@yahoo.com.tw (C.-T.L.); kllaichiayi@yahoo.com.tw (K.-L.L.); crashbug1982@gmail.com (K.-T.T.); cwtseng@vghtc.gov.tw (C.-W.T.); gtim5555@yahoo.com (W.-N.H.); dr.yihsing@gmail.com (Y.-H.C.); 2 Department of Medical Education, Taichung Veterans General Hospital, Taichung 40705, Taiwan; 3 Department of Nuclear Medicine, Taichung Veterans General Hospital, Taichung 40705, Taiwan; dianayjlin@gmail.com (Y.-C.L.); wy1962@gmail.com (W.-Y.L.); 4 Department of Public Health, China Medical University, Taichung 40447, Taiwan; 5 Department of Medical Research, Taichung Veterans General Hospital, Taichung 40705, Taiwan; 6 Institute of Biomedical Science and Rong Hsing Research Center for Translational Medicine, National Chung Hsing University, Taichung 402, Taiwan; 7 Faculty of Medicine, National Yang-Ming University, Taipei 11221, Taiwan; 8 Department of Pathology and Laboratory Medicine, Taichung Veterans General Hospital, Taichung 40705, Taiwan; mewen@vghtc.gov.tw; 9 Department of Medical Imaging and Radiological Sciences, Central Taiwan University of Science and Technology, Taichung 406053, Taiwan

**Keywords:** systemic lupus erythematosus, lupus nephritis, glomerulonephritis, gallium scan, scintigraphy, renal biopsy

## Abstract

Background: Lupus nephritis (LN) often lead to end-stage renal disease in systemic lupus erythematosus patients. This study aimed to investigate the clinical application of renal gallium-67 scans for determining renal histological parameters in LN patients. Methods: Between 2006 and 2018, 237 biopsy-proven and 35 repeat biopsies LN patients who underwent renal gallium scans before or after biopsy were included for analysis. The classification and scoring of LN were assessed according to the International Society of Nephrology/Renal Pathology Society. A delayed 48-h gallium scan was performed and interpreted by semiquantitative methods using left kidney/spine (K/S) ratio. The renal histological results were compared with gallium uptake. Results: Out of 237 participants, 180 (76%) had proliferative LN. Baseline gallium left K/S ratio was significantly higher in class IV LN as compared to class III (median (interquartile range, IQR): 1.16 (1.0–1.3), 0.95 (0.9–1.1), respectively, *p* < 0.001). Furthermore, changes in gallium uptake between two biopsies were positively correlated with changes activity index (r = 0.357, *p* = 0.035), endocapillary hypercellularity (r = 0.385, *p* = 0.032), and neutrophils infiltration (r = 0.390, *p* = 0.030) in renal pathology. Conclusions: Renal gallium uptake is associated with active inflammation in LN. Changes in renal gallium uptake positively correlated with changes in activity index in renal pathology.

## 1. Introduction

Lupus nephritis (LN) occurs in approximately sixty percent of patients with systemic lupus erythematosus (SLE) [1]. The goals for managing patients with lupus nephritis include early diagnosis and proper therapy, which may prevent irreversible renal damage [2]. Therefore, a kidney biopsy should be performed in LN patients who develop overt proteinuria or renal insufficiency, as it is essential in assessing disease activity and guiding treatment [3,4]. The recommendation for monitoring LN includes urinalysis, including urine protein/creatinine ratio, serum creatinine, complement 3 (C3), C4 levels, and anti-DNA levels [5]. However, some patients with the quiescent disease may develop a recurrent emergence of a new elevation in serum creatinine, and/or worsening proteinuria despite treatment. In this event, a repeat biopsy may help assess the renal inflammatory activity and chronic damage, which could help guide subsequent treatment. Renal biopsy is an invasive diagnostic procedure, and bleeding complications were significantly higher in patients with serum creatinine above 2 mg/dL (2.1 versus 0.4 percent) [6]. Therefore, detecting active disease using a non-invasive tool may ensure the levels of immunosuppressants aretailored to the disease activity. 

Gallium-67 scintigraphy has been used for decades to evaluate interstitial nephritis [7,8]. After gallium injection, it may bind to lactoferrin in neutrophils, be taken up by lysosomes in mono-nuclear phagocytes, bind directly to the lymphocyte membrane, and be transported to sites of inflammatory glomeruli. Gallium-67 scintigraphy has also been described as a helpful tool in evaluating LN activity [9,10], and can also be used to monitor LN due to its relationship with disease activity, and its potential as a diagnostic alternative to renal biopsy [9,10,11]. Previously, our group developed a semiquantitative method for gallium renal imaging, which showed that the left kidney-to-spine ratio (K/S ratio) determined using a semiquantitative method had a better correlation with renal biopsy results than those obtained by visual grading [11]. However, no studies have evaluated the correlation between changes in renal gallium uptake and the changes in renal pathology findings. 

In this hospital-based study, we aimed to investigate the clinical value of the change of renal gallium-67 scintigraphy uptake using the left K/S ratio for evaluating the change of renal histological parameters in LN patients in LN patients with repeated biopsies.

## 2. Materials and Methods

### 2.1. Participants

Between January 2006 and December 2018, a hospital-based observational study was conducted to retrospectively review 237 biopsy-proven LN patients, including 35 patients who received repeat biopsies. All patients underwent single or repeated renal gallium scans within a period of 30 days before or after the biopsy (with a median of 4 days with standard deviation of 11.97). The classification and scoring of LN and details of renal histology exams were recorded. A delayed 48-h gallium scan was performed and interpreted by semiquantitative methods. The renal histological results were compared with gallium scan parameters. This study was conducted in compliance with the Helsinki Declaration, and approval by the Institutional Review Board of Taichung Veterans General Hospital was obtained (CE18210B). As all data were de-identified before analysis, the requirement for informed consent from the participants was waived.

### 2.2. Clinical Parameters

Clinical data included daily urine protein, serum creatinine, estimated glomerular filtration rate (eGFR), anti-double strand DNA antibody (dsDNA Ab), C3, and C4 were collected before renal biopsy. Daily urine protein was measured by spot urine protein and creatinine ratio. Estimated GFR was calculated using the Modification of Diet in Renal Disease equations [12]. Whether or not a second kidney biopsy was deemed necessary depended on the treating clinician’s decision.

### 2.3. Renal Histology

All patients underwent percutaneous renal biopsy, which was performed by experienced nephrologists under ultrasonic guidance. The classification and scoring of LN were assessed according to the International Society of Nephrology/Renal Pathology Society [13]. The activity index (AI) and chronicity index (CI) were calculated based on the system devised by Austin and colleagues [14]. This renal pathology grading system (AI and CI) has been widely used for several decades and has been recognized as a predictor of renal outcomes [15,16]. In this system, AI is defined as the sum of the individual scores of 6 features (glomerular cell proliferation, leukocyte exudation, fibrinoid necrosis/karyorrhexis, cellular crescents, hyaline deposits, and interstitial inflammation) considered to represent measures of active lupus nephritis. Chronicity index is defined as the sum of the individual scores of four features (glomerular sclerosis, fibrous crescents, tubular atrophy, and interstitial fibrosis), which considered to represent measures of chronic irreversible lupus nephritis. Each individual component is scored on a scale of 0 to 3+ (0, absent; 1+, mild; 2+, moderate; 3+, severe). The maximum AI value is 18 points, and the maximum CI value is 12 points. The World Health Organization classification, AI, and CI were determined by two pathologists, who were blind to the results of clinical parameters and gallium renal scans.

### 2.4. Gallium-67 Renal Scintigraphy

Gallium scintigraphy was performed within 30 days before or after the renal biopsy. Forty-eight hours after the injection of 111 MBq (3 mCi) of 67Ga citrate, static posterior abdominal scintigrams were obtained using a large-field-of-view camera with a medium-energy, parallel-hole collimator (ECAM; Siemens, Munich, Germany). Three 20% windows were set at 93,184 and 296 keV. Residual bowel activity is a potential source of error on the gallium image, and thus bowel preparation was performed in all patients before imaging. If the kidney image was overlapped by bowel activity at forty-eight hours, the seventy-two hours after administration of the radiotracer images were used instead. The SPECT volume session included the abdomen with an axial field of view of 40 cm, followed by a low dose CT of the same correlative territory. SPECT images were obtained with a non-circular orbit with step and shoot acquisition, obtaining 64 images of 50 s each in a 128 × 128 pixels matrix. SPECT data were reconstructed using a three-dimensional iterative algorithm. CT data information were used for attenuation correction and anatomical information. No contrast medium was injected during the examination.

### 2.5. Semi-Quantitative Method of Gallium Renal Scintigraphy

**Figure 1:**
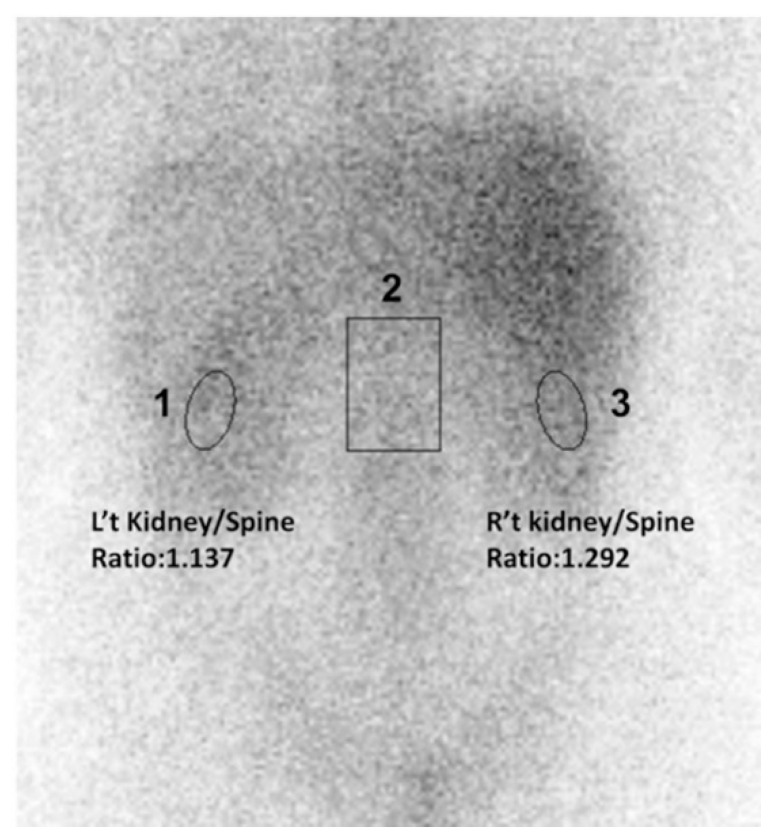
Semiquantitative measurement calculated by kidney/spine ratio from 67Ga renal image (posterior view) in a patient with lupus nephritis. ROIs on scintigraphy image: ROI 1: left kidney; ROI 2: spine; ROI 3: Right kidney. ROI: region of interest.

For semi-quantitative analysis of gallium uptake in both kidneys, regions of interest (ROIs) were drawn over both kidneys and the adjacent spine (Figure 1). To minimize subjective bias, we set each ROIs of images by the following rules. First, the ROI of kidneys should locate in the middle or inferior parts of visible renal parenchyma to avoid interference of tracer-avid organs such as liver, spleen, and bowels. Second, the average pixel size of renal ROI is 100. The ROI of spines should locate in the adjacent spines, which are longer than one vertebral height to avoid single structural or degenerative deformities and is no wider than a vertebral width to exclude the structure outside the vertebral body. The average pixel size of spine ROI is 500. Third, two experienced technicians in one workstation did all procedures. For each ROI, a value of mean counts per pixel was obtained for data analysis. The uptake ratios between ROIs were calculated and expressed as “kidney/spine ratio” (K/S ratio).

### 2.6. Patient and Public Involvement

We did not involve patients or the public in our work.

### 2.7. Statistical Analysis

The demographic data of continuous parameters are shown as mean ± standard deviation, and for categorical variables as the number of patients. Chi-Square test and Kruskal-Wallis test were used to perform unadjusted comparisons among patients with various classes of LN. Factors associated with renal gallium uptake were determined using multivariable linear regression. Comparisons of variables of first vs. subsequent renal biopsies were performed by Wilcoxon signed rank and McNemar test. Correlation between changes of gallium uptake between two renal biopsies and clinical/histological variables were performed by Spearman’s correlation. All data were analyzed using the Statistical Package for the Social Sciences (SPSS), version 22.0. Significance was set at *p* < 0.05.

## 3. Results

### 3.1. Demographic Data of Enrolled LN Patients by Pathological Classification

A total of 237 participants (195 women, 88.6%) were included in the study. Demographic, clinical, histological features, and gallium left kidney/spine ratio (left K/S ratio) are shown in Table 1. Out of 237 participants, 170 (72%) had proliferative LN (class III and class IV). The activity index of class IV was significantly higher than class III and class V (median (inter-quartile range, IQR): 8 (5.0–10.3), 3 (1.0–3.8), 0 (0–2), respectively, *p* < 0.001). Gallium left K/S ratio was significantly higher in class IV LN as compared to class III (1.16 (1.0–1.3), and 0.95 (0.9–1.1), respectively, *p* < 0.001), but not class V.

### 3.2. Factors Associated with Renal Gallium Uptake

**Figure 2:**
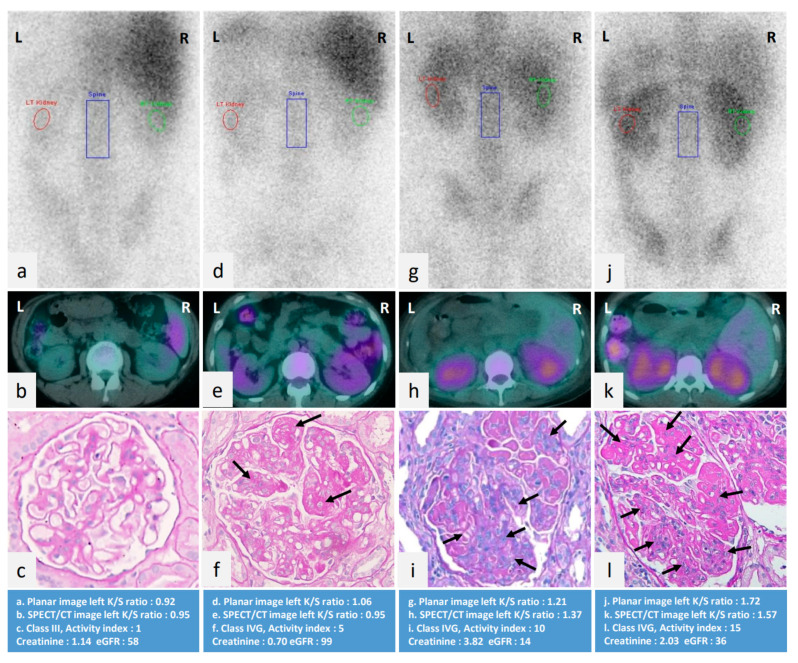
Renal gallium planar view (**a**,**d**,**g**,**j**) and SPECT/CT (**b**,**e**,**h**,**k**) images of four different LN patients from low to high activity index scoring in renal histopathology (**c**,**f**,**i**,**l**). (**a**–**c**) A 34-year-old LN female patient with activity index 1 in pathology, left K/S ratio 0.92 in gallium planar image; (**d**,**e**) A 40-year-old LN female patient with activity score 5 in pathology, left K/S ratio 1.06 in gallium planar image; (**g**–**i**) A 41-year-old LN female patient with activity score 10 in pathology, left K/S ratio 1.21 in gallium planar image; (**j**–**l**) A 14-year-old LN female patient with activity score 15 in pathology, left K/S ratio 1.72 in gallium planar image. This figure revealed that a trend of a higher degree of gallium uptake calculated by left K/S ratio, the greater degree of activity index score and endocapillary hypercellularity (arrows) L: left side; R: right side; SPECT/CT: single photon emission computed tomography; LN: lupus nephritis; K/S ratio: kidney/spine ratio.

The univariate and multivariate linear regression analysis in Table 2 shows the factors associated with left K/S ratio. Univariate linear regression demonstrated that daily urinary protein, eGFR, activity index, cellular crescents, fibrinoid necrosis/karyorrhexis, endocapillary hypercellularity, subendothelial hyaline deposits, leukocyte infiltration, and interstitial inflammation were significantly associated with renal gallium uptake. In multivariate analysis, daily urinary protein, activity index, endocapillary hypercellularity, and interstitial inflammation independently correlated with gallium uptake left K/S ratio. Figure 2 illustrates that the higher degree of gallium uptake calculated by left K/S ratio, the greater degree of activity index in renal histopathology.

### 3.3. Comparisons of Clinical Variables of LN Patients Receiving Repeat Renal Biopsies

Thirty-five patients received a second kidney biopsy and renal gallium scan (Table 3). The mean scores of activity index, fibrinoid necrosis/karyorrhexis, and endocapillary hypercellularity in the second biopsy were significantly decreased compared to the first renal biopsy. The creatinine level, C4, mean scores of chronicity index, glomerular sclerosis, tubular atrophy, and interstitial fibrosis in the second biopsy were significantly increased compared to the first renal biopsy. The K/S ratio was non-significantly decreased. 

### 3.4. Correlation between Changes of Renal Gallium Uptake and Changes of Clinical Variables

**Figure 3:**
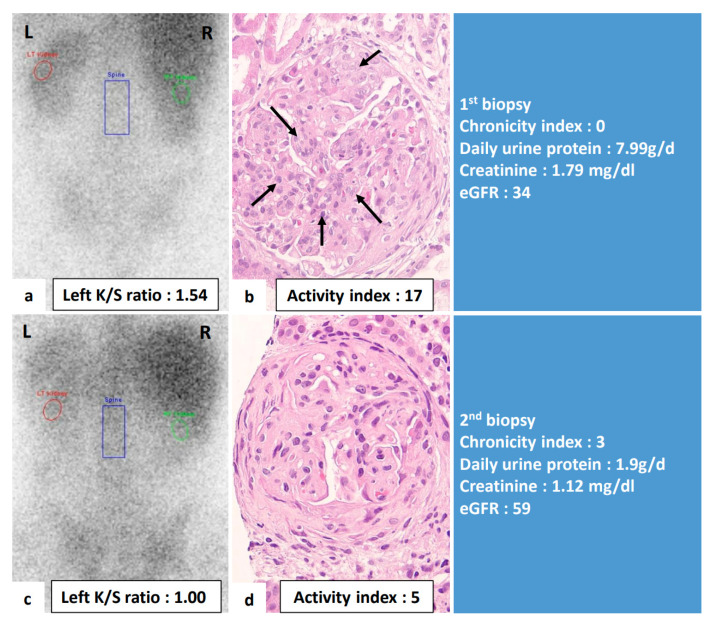
Renal gallium scan and renal histopathologic images in a 36-year-old female with lupus nephritis at diagnosis and one year after immunosuppressant treatment. This figure demonstrated that the change of renal gallium uptake was positively correlated with the change of the inflammatory status in renal histology and clinical proteinuria. (**a**,**b**) At diagnosis, renal pathology active index score was 17. The Left K/S ratio was 1.54. Daily urine protein was 7.99 g/d. (**c**,**d**) One year after immunosuppressant treatment, the activity index decreased to 5, left K/S ratio decreased to 1.00. Daily urine protein decreased to 1.9 g/d. L: left side; R: right side; K/S ratio: kidney/spine ratio; arrows: endocapillary hypercellularity.

**Figure 4:**
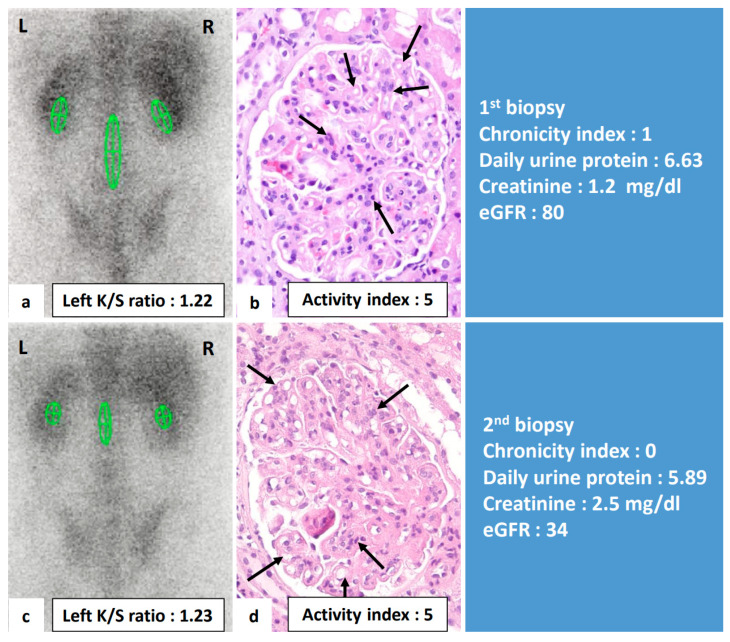
Renal gallium scan and renal histopathologic images of a 22-year-old male with lupus nephritis at diagnosis and two years after immunosuppressant treatment. This figure demonstrated that persistent undiminished gallium uptake was compatible with persistent active inflammation in renal histology and clinical proteinuria. (**a**,**b**) At diagnosis, renal pathologic active index score was 5. The Left K/S ratio was 1.22. Daily urine protein was 6.63 g/d. (**c**,**d**) Two years after immunosuppressant treatment, the activity index remained 5, left K/S ratio was 1.23 without diminished uptake, daily urine protein persisted at a high level of 5.89 g/d. L: left side; R: right side; K/S ratio: kidney/spine ratio; arrows: endocapillary hypercellularity.

To further explore the clinical significance of gallium uptake, we analyzed the change of gallium uptake and the change of variate of clinical parameters in individual patients. Table 4 shows the results of analyses performed to determine whether change of gallium uptake was sensitive to changes of clinical and pathologic parameters. A change in gallium uptake between two biopsies were positively correlated with changes of daily urine protein (*r* = 0.768, *p* < 0.001), changes of activity index scores (*r* = 0.357, *p* = 0.035), endocapillary hypercellularity (*r* = 0.385, *p* = 0.032), and leukocyte infiltration (*r* = 0.390, *p* = 0.030). Change of creatinine level and eGFR was not significantly correlated with change of left K/S ratio. Figure 3 demonstrated an example of this study that the changes in gallium uptake calculated by left K/S ratio between two biopsies were positively correlated with changes of daily urine protein and activity index score, but not chronicity index, in renal pathology. Figure 4 demonstrates an example where no significant change of renal gallium uptake between two biopsies was observed and no change in the activity index score in the renal pathology.

## 4. Discussion

This work found that the degree of renal gallium uptake calculating by left K/S ratio was significantly higher in LN class IV than class III. However, not class V. Lt K/S ratio of class V is slightly significantly higher than class III. The activity index of class III was non-significantly higher than class V. From the above result, we could see a trend that renal gallium uptake is associated with active inflammation in LN, but is not sensitive, especially compared to different patients. This is a relatively small number of statistics. Another concern is that eGFR may affect the K/S ratio and reduce its sensitivity in detecting disease activity/inflammation. From Table 1, eGFR is higher in Class III and V groups than in Class IV. The K/S is correspondingly lower in Class III and V groups. Therefore, the gallium uptake and a higher K/S ratio might reflect slower renal clearance of the isotope in patients with reduced excretory renal function. From the previous studies, after administration of Ga-67, excretion of 15% to 25% of the dose occurs through the kidneys in the first 24 h, with the bowel becoming the major route of excretion after that [17]. The gallium image was used after 48 h of gallium administration to diminish the impact of renal function on gallium uptake. Besides, we did not use the absolute kidney gallium uptake value but use the spine as a reference. In theory, if a patient has a poor renal function, higher gallium accumulation in the whole body should be expected, particularly in the liver, spleen, and bones. If a patient has a higher renal inflammation, more gallium uptake is thus expected in the kidney, and this could still be demonstrated by the gallium uptake of Kidney/Spine ratio. By using the K/S ratio, the impact of renal function on gallium uptake might be minimized.

To explore whether eGFR impacts renal gallium uptake, we conducted a linear regression analysis (Table 2). From univariate linear regression analysis, eGFR is associated with left K/S ratio. In the multivariate analysis 1, the activity index is significantly associated with left K/S ratio, and eGFR is not associated with left K/S ratio. 

In the pooling analysis of all 35 patients with repeated biopsy, the activity index in the second biopsy was significantly lower than the first biopsy, but the K/S ratio was non-significantly decreased. This may be associated with the increased creatinine level in the second biopsy, resulting in slightly increased gallium uptake. Besides, this was a comparison of all patients together and unable to show the difference between the two biopsies for each patient. Table 4 demonstrated that the changes in gallium uptake between the first and the second biopsies were positively correlated with the changes in histological activity index, the scores of endocapillary hypercellularity, and leukocyte infiltration. The changes in creatinine and eGFR was not correlated with change in gallium uptake. The results shed light on a novel non-invasive assessment for residual renal inflammation in LN patients that may have potential as a substitute for traditional histology exams. As discussed above, a change in creatinine may be potentially confounded by the fact that patients with higher disease activity (predominantly in first biopsy group), which may increase K/S ratio, showed lower serum creatinine (which may decrease K/S ratio). 

In this study, we set smaller ROIs of kidney instead of whole kidney, which we used in our previous study in 2010 [11]. There are some reasons we considered. First, strong liver gallium uptake may physiologically mask subtle changes of kidney. Second, the renal collecting systems, such as calyces and pelvis with relative low gallium uptake might underestimate mean counts per pixel if whole kidney was included. Our limitation is the lack of validation between the data of whole kidney ROIs and smaller ROIs.

In 1978, Barry et al. reported that a SLE patient with renal biopsy-proved LN could be evaluated by renal gallium scan. Moreover, scintigraphy of kidneys showed significantly decreased inflammation after high-dose glucocorticoid therapy [18]. BAKIR et al. suggested a high correlation between gallium visualization of the kidneys and active lupus nephritis [19]. They also found that hypertension, nephrotic range proteinuria, and progressive azotemia were more frequently encountered when the gallium scan result was positive. Absolute quantitative and semi-quantitative measurements by gallium scan exhibited good correlation with the activity index of renal biopsy [20]. However, the value of the renal gallium study in evaluating LN following therapies has never been explored. To the best of our knowledge, this study is the first to evaluate the correlation of changes in renal gallium uptake with changes in pathologic parameters based on repeated renal biopsies. In our data, the mean activity index in the second biopsy was significantly decreased, and mean chronicity index was significantly increased compared to the first biopsy. However, no notable changes in class were found in our results. The above findings are compatible with the results of repeat biopsy for LN in previous reports [20,21,22,23]. 

Conventional laboratory markers of urine protein creatinine ratio, creatinine clearance, anti-dsDNA, and complement levels have limitation for monitoring LN, since they might be underpowered to distinguish renal inflammation activity from irreversible chronic renal damage in LN [24]. This is a critical issue, especially when LN patients develop a new onset or progressively elevated serum creatinine or proteinuria during disease monitor. In this scenario, the aggressive immunosuppressant treatment is suitable if active renal inflammation is present but may be harmful to chronic damage without inflammation process. Conventional laboratory markers could tell clinicians the worsening renal function or proteinuria severity but not correlated with active renal inflammation. We currently rely on repeated renal biopsy to differentiate these two processes. However, the renal histology exam is an invasive procedure, which makes it unsuitable for serial monitoring. In addition, there is no universal agreement regarding the indications for repeated renal biopsy in LN patients. It has been advocated that protocol biopsies could be performed at 6 months or after 1-2 years following therapies in stable patients to confirm the response to induction therapy [22,25,26,27,28,29,30] or to verify the efficacy of maintenance therapy [21,31,32,33]. Moreover, repeated biopsies could also provide additional valuable information in LN patients with renal flares [20,23,34,35,36]. Repeated renal biopsy may offer crucial information for challenging LN cases to guide the intensity of treatment in patients with quiescent LN and in those with lupus flare-up. However, due to their invasive nature, renal biopsies can also be physically painful and emotionally distressing. Furthermore, the procedure may be even more risky and challenging in LN patients whose renal function is deteriorating, and who have a thin renal cortex or severe hypertension despite medication, as there is an elevated risk of hemorrhagic complications following renal biopsy [37]. In this scenario, renal gallium scan may have value as a potential alternative to renal biopsy for interrogating the degree of renal inflammation in LN, which could, in turn, facilitate better therapeutic decisions. 

A study by A. Alsuwaida et al. demonstrated that the activity index in the second renal biopsy, not the first biopsy, may predict a poor renal outcome. The 10-year renal survival rate was 100% for those with an activity index of 0; 80% for those with an activity index of 1 or 2; and 44% for those with an index of >2 on the second biopsy, regardless of remission status [31]. Similar findings were also shown by Hill GS et al. [25]. They found the rate of serum creatinine doubling was higher among LN patients with persistent active lesions, especially endocapillary proliferation and interstitial inflammation, compared to those with a resolution of active inflammation in the second biopsy. Our study found that changes in gallium uptake measured by left K/S ratio were correlated with changes in active histopathological parameters, including endocapillary proliferation and leukocyte infiltration. These findings indicate that a follow-up renal gallium scan in patients with repeated renal biopsies provided essential information related to kidney inflammation following treatment. In the LN case in our study, shown in Figure 4, the repeated renal biopsy two years after immunosuppressant treatment showed persistent active lesion, especially endocapillary hypercellularity. The repeated renal gallium uptake also remains unchanged without resolution. Finally, the patient’s renal function deteriorated, and he received hemodialysis 11 years after diagnosis. Future outcome-based studies, guided by subsequent gallium scan studies, may provide more insights into the potential application of scintigraphy in clinical practice.

It remains unclear why gallium uptake in a positive renal scan was observed in active lupus nephritis patients. This study and other findings indicate that a high degree of renal gallium uptake was observed in patients with high renal activity [19]. In proliferative LN, affected glomeruli display endocapillary proliferation. These lesions are characterized by the deposition of immunoglobulins, complements, and marked influx of pro-inflammatory leukocytes, monocytes, macrophages, and suppressor/cytotoxic T cells, as well as neutrophils infiltration [38]. After gallium injection, one-fourth of the injected dose is excreted by the kidneys within 24 h [17]. Gallium-67 may bind to lactoferrin in neutrophils, are taken up by lysosomes in mononuclear phagocytes, bind directly to the lymphocyte membrane, and transported to sites of inflammatory glomeruli [39]. In this study, endocapillary proliferation and interstitial inflammation were independently positively associated with gallium uptake. The gallium-67 uptake from the proliferated endothelial cells and interstitial neutrophile cells in LN patients might explain the above phenomenon in this study. More basic research may be needed to confirm this finding.

In many areas of clinical practice, gallium scanning has been superseded by PET imaging using FDG and Tc-99 scans, which have several advantages, including a shorter delay between injection of the radioisotope and completing the imaging. However, the patient’s steroids are likely to interfere with PET interpretation, especially during the high activity LN patient who received high-dose steroid. Technetium 99m (99mTc)-HMPAO is an agent that complexes avidly with polymorphonuclear leukocytes and has been used to evaluate the areas of acute inflammation. A limitation of this procedure is that 70–80 mL of blood must be drawn from the patient for this technique. This may increase the possible need for a blood transfusion in patients already experiencing anemia, especially in renal insufficiency patients under immunosuppressant agents.

Our study found that the gallium K/S ratio in the right kidney was higher than that in left side in every LN class (Table 1). This is most likely due to the high gallium radioactivity in the normal liver, which may interfere with the calculation of the right K/S ratio. In order to avoid this, we meticulously calculated the left side K/S ratio for subsequent analysis. This concept was initially explored in our previous study on renal gallium scan for evaluating lupus nephritis [11], and the findings of the present study further confirm this interference effect. 

Many biomarkers based on laboratory tests have been explored as possible non-invasive methods of evaluating LN [40]. Unfortunately, very few biomarker studies have been done at the time of renal biopsy, and no studies have evaluated the correlation between the change in biomarkers and the change in repeat renal biopsy [40]. This study provides valuable image information between repeated renal biopsies in LN patients. 

There were several limitations in the study. First, changes in excretory renal function have an effect on gallium uptake, which may confound some of the findings presented in this work, and limit the sensitivity of this technique in detecting small changes active inflammation. Second, subjective bias in the positioning of the ROI might lead to biased and inconsistent analysis between different patients. Third, the relatively long period of 30 days between kidney biopsy and renal gallium scan used in this study may have implications for how well the scintigraphy findings reflect the histopathological features seen on biopsy in individual patients. Fourth, this was a retrospective study with a limited sample size, and only one ethnic group (East Asians) was included. The results may not be extrapolated to other populations. Further studies using a larger cohort are needed to confirm our findings. Fifth, renal survival and patient survival were not assessed in this study. A recent study indicated that activity scores in LN histology were associated with end-stage renal disease [41]. Whether the degree of renal gallium uptake has a predicting role on renal survival in LN patients needs further study. Sixth, renal gallium uptake cannot distinguish inflammation from other conditions, such as infection (e.g., acute pyelonephritis, renal abscess) and tumor (e.g., lymphoma) [18,42,43]. Clinicians should incorporate other clinical assessments and imaging modalities to reach the final diagnosis if necessary. Finally, it remains unclear how immunosuppressants with different mechanisms of action influence gallium renal uptake. Prospective intervention studies are needed to address this issue.

## 5. Conclusions

This study demonstrated that renal gallium uptake is associated with pathologic active inflammation in LN but not chronicity damage lesion. The association may be impacted by renal function. Changes in renal gallium uptake positively correlated with changes in activity index in renal pathology. Currently, renal biopsy remains the standard evaluation for lupus nephritis inflammatory activity. However, as a non-invasive tool, renal gallium scan may add value to the personalized care of patients with LN, particularly those with a high risk for biopsy. More researches with large cohorts are needed for further validation.

## Figures and Tables

**Table 1:** Basic demographic data of 237 lupus nephritis patients by histological classes.

	Class I & II (*n* = 7)	III (*n* = 36)	IV (*n* = 134)	V (*n* = 60)	*p* Value
Age (years)	42.0 (33.0–43.0)	31.5 (25.3–40.0)	32.0 (25.0–41.0)	31.5 (26.0–39.5)	0.586
Female gender	7 (100.0%)	30 (83.3%)	108 (80.6%)	50 (83.3%)	0.606
Laboratory data					
Daily urine protein (gram)	0.7 (0.1–1.8)	1.9 (0.9–2.4)	3.7 (2.1–5.8)	2.3 (1.1–7.0)	<0.001 **^‡§||^
Creatinine (mg/dL)	0.7 (0.5–0.8)	0.9 (0.6–1.2)	1.4 (0.9–2.9)	0.8 (0.7–1.1)	<0.001 **^‡||††^
eGFR (mg/mL)	97.5 (91.0–139.2)	85.1 (63.5–118.4)	51.7 (22.1–79.9)	84.3 (60.7–117.1)	<0.001 **^‡||††^
Anti-dsDNA (WHO units/mL)	248.7 (166.5–477.0)	174.3 (71.2–353.0)	265.4 (125.5–464.1)	130.4 (35.6–277.6)	<0.001 **^††^
C3 (mg/dL)	78.2 (71.3–90.5)	77.4 (64.5–94.0)	54.6 (39.4–66.7)	63.8 (46.5–89.9)	<0.001 **^||††^
C4 (mg/dL)	15.1 (4.1–20.9)	14.2 (10.6–22.1)	10.2 (6.3–17.6)	11.7 (6.5–23.5)	0.045 *^||^
Renal pathology					
Activity index	0 (0–1)	3 (1.0–3.8)	8 (5.0–10.3)	0 (0–2)	<0.001 **^‡||††^
Cellular crescents	0 (0–0)	0 (0–0)	2 (0–2)	0 (0–0)	<0.001 **^‡||††^
Fibrinoid necrosis/ Karyorrhexis	0 (0–0)	0 (0–2)	2 (0–2)	0 (0–0)	<0.001 **^‡††^
Endocapillary hypercellularity	0 (0–0)	1 (0–1)	2 (2–3)	0 (0–1)	<0.001 **^‡||††^
Subendothelial hyaline deposits	0 (0–0)	0 (0–0)	1 (0–2)	0 (0–0)	<0.001 **^‡||††^
Leukocyte infiltration	0 (0–0)	0 (0–0)	1 (0–1)	0 (0–0)	<0.001 **^||††^
Interstitial inflammation	0 (0–1)	0 (0–1)	1 (0–2)	0 (0–1)	<0.001 **^||††^
Chronicity Index	0 (0–1)	3 (0–3)	1 (0–4)	1 (0–2.8)	0.159
Glomerular sclerosis	0 (0–0)	1 (0–1)	1 (0–1)	0 (0–1)	0.011 *
Tubular atrophy	0 (0–0)	1 (0–1)	0 (0–1)	0 (0–1)	0.157
Interstitial fibrosis	0 (0–1)	1 (0–1)	0 (0–1)	0 (0–1)	0.656
Fibrous crescent	0 (0–0)	0 (0–0)	0 (0–0)	0 (0–0)	0.155
Left K/S ratio	0.910 (0.8–1.2)	0.950 (0.9–1.1)	1.16 (1.0–1.3)	1.040 (1.0–1.2)	<0.001 **^||^^¶^
Right K/S ratio	0.990 (0.9–1.4)	0.995 (0.9–1.2)	1.220 (1.1–1.4)	1.080 (1.0–1.2)	<0.001 **^||††^

* *p* < 0.05, ** *p* < 0.01; Post hoc analysis. ^‡^ I/II vs. IV *p* < 0.05; ^§^ I/II vs. V, *p* < 0.05; ^||^ III vs. IV, *p* < 0.05; ^¶^ III vs. V, *p* <0.05; ^††^ IV vs. V, *p* < 0.05. eGFR: estimated glomerular filtration rate, anti-dsDNA: anti-double-stranded DNA antibody, C3: complement 3, C4: complement 4, SLEDAI: systemic lupus erythematosus disease activity index, K/S ratio: kidney-to-spine ratio.

**Table 2:** Linear regression of clinical and histological parameters associated with left K/S ratio.

	Univariate		Multivariate 1		Multivariate 2		Multivariate 3	
	B (95%CI)	*p* Value	B (95%CI)	*p* Value	B (95%CI)	*p* Value	B (95%CI)	*p* Value
Age (years)	−0.003 (−0.005, 0.000)	0.061						
Gender								
Female	Reference							
Male	0.004 (−0.082, 0.088)	0.941						
Laboratory data								
Daily urine protein (gram)	0.022 (0.015, 0.030)	<0.001 **	0.019 (0.011, 0.026)	<0.001 **	0.018 (0.010, 0.026)	<0.001 **	0.018 (0.010, 0.025)	<0.001 **
Creatinine (mg/dL)	0.018 (−0.001, 0.037)	0.067						
eGFR (mg/mL)	−0.001 (−0.002, 0.000)	0.016 *	0.000 (−0.001, 0.001)	0.752	0.000 (−0.001, 0.001)	0.994		
Anti-dsDNA (WHO units/mL)	0.000 (0.000, 0.000)	0.350						
C3 (mg/dL)	−0.001 (−0.002, 0.000)	0.072						
C4 (mg/dL)	−0.001 (−0.003, 0.002)	0.565						
Renal pathology								
Activity index	0.021 (0.015, 0.028)	<0.001 **	0.018 (0.010, 0.025)	<0.001 **				
Cellular crescents	0.051 (0.026, 0.070)	<0.001 **			0.008 (−0.023, 0.030)	0.618		
Fibrinoid necrosis/Karyorrhexis	0.036 (0.016, 0.057)	<0.001 **			0.017 (−0.008, 0.041)	0.185		
Endocapillary hypercellularity	0.072 (0.045, 0.098)	<0.001 **			0.025 (−0.017, 0.066)	0.241	0.041 (0.011, 0.070)	0.008 **
Subendothelial hyaline deposits	0.082 (0.046, 0.118)	<0.001 **			0.018 (−0.029, 0.065)	0.456		
Leukocyte infiltration	0.090 (0.040, 0.141)	<0.001 **			−0.014 (−0.071, 0.044)	0.643		
Interstitial inflammation	0.094 (0.059, 0.130)	<0.001 **			0.054 (0.013, 0.095)	0.010 *	0.061 (0.023, 0.100)	0.002 **
Chronicity Index	−0.002 (−0.015, 0.010)	0.694						
Glomerular sclerosis	−0.020 (−0.054, 0.014)	0.244						
Tubular atrophy	−0.009 (−0.046, 0.029)	0.648						
Interstitial fibrosis	−0.001 (−0.039, 0.036)	0.848						
Fibrous crescent	0.049 (−0.077, 0.175)	0.442						

* *p* < 0.05, ** *p* < 0.01. Multivariate 1 Adjust R^2^ = 0.219; Multivariate 2 Adjust R^2^ = 0.217; Multivariate 3 Adjust R^2^ = 0.225. eGFR: estimated glomerular filtration rate; anti-dsDNA: anti-double-stranded DNA antibody; C3: complement 3; C4: complement 4; SLEDAI: systemic lupus erythematosus disease activity index; K/S ratio: kidney-to-spine ratio.

**Table 3:** Comparisons of clinical, pathological parameters and gallium uptake between two renal biopsies in 35 SLE patients.

	First Biopsy	Second Biopsy	*p* Value
Age (years)	26.0 (21.0–36.0)	30.0 (24.0–37.0)	<0.001 **
Daily urine protein (gram)	3.8 (1.8–6.7)	3.9 (1.0–5.6)	0.600
Creatinine (mg/dL)	1.0 (0.8–1.7)	1.1 (0.8–2.7)	0.013 *
eGFR (mg/mL)	74.1 (38.3–95.3)	58.1 (22.5–96.4)	0.072
Anti-dsDNA (WHO units/mL)	148.75 (95.8–409.1)	141.4 (37.0–364.7)	0.135
C3 (mg/dL)	56.9 (41.9–83.8)	70.4 (48.9–87.3)	0.109
C4 (mg/dL)	10.5 (5.7–13.5)	16.8 (6.2–22.3)	0.020 *
Lupus nephritis category			0.931
I & II	2 (5.7%)	2 (5.7%)	
III	3 (8.6%)	3 (8.6%)	
IV	22 (62.9%)	23 (65.7%)	
V	8 (22.9%)	7 (20.0%)	
Activity index	6.0 (1.0–9.0)	2.0 (0.3–6.8)	0.028 *
Cellular crescents	0 (0–2)	0 (0–0)	0.218
Fibrinoid necrosis/ Karyorrhexis	2.0 (0.0–2.0)	0 (0–0)	0.028 *
Endocapillary hypercellularity	2 (0–3)	1 (0–3)	0.045 *
Subendothelial hyaline deposits	0 (0–2)	0 (0–1)	0.301
Leukocyte infiltration	0 (0–1)	0 (0–1)	0.120
Interstitial inflammation	1.0 (0.0–1.0)	1.0 (0.0–1.0)	0.655
Chronicity Index	1 (0–2)	3.0 (1.0–7.0)	<0.001 **
Glomerular sclerosis	0 (0–1)	1 (0–3)	<0.001 **
Tubular atrophy	0 (0–0)	1 (0–2)	0.001 **
Interstitial fibrosis	0 (0–1)	1 (0–2)	0.001 **
Fibrous crescent	0 (0–0)	0 (0–0)	0.317
Left K/S ratio	1.140 (1.0–1.4)	1.060 (1.0–1.3)	0.241
Right K/S ratio	1.210 (1.1–1.4)	1.120 (1.0–1.3)	0.225

* *p* < 0.05, ** *p* < 0.01. eGFR: estimated glomerular filtration rate; anti-dsDNA: anti-double-stranded DNA antibody; C3: complement 3, C4: complement 4; K/S ratio: kidney-to-spine ratio.

**Table 4:** Correlation of changes in clinical, pathologic parameters and change of renal gallium uptake in 35 SLE patients.

Changes in Variables	Change in Left K/S Ratio	
	r_s_	*p* Value
∆Daily urine protein (gram)	0.768	<0.001 **
∆Creatinine (mg/dL)	0.045	0.796
∆eGFR (mg/mL)	−0.192	0.270
∆Anti-dsDNA (WHO units/mL)	0.768	0.348
∆C3 (mg/dL)	0.027	0.888
∆C4 (mg/dL)	0.022	0.902
∆Activity Index	0.357	0.035 *
∆Cellular crescent	0.258	0.161
∆Fibrinoid necrosis/ Karyorrhexis	0.324	0.075
∆Endocapillary hypercellularity	0.385	0.032 *
∆Subendothelial hyaline deposits	0.253	0.017
∆Leukocyte infiltration	0.390	0.030 *
∆Interstitial inflammation	0.300	0.101
∆Chronicity Index	−0.106	0.543
∆Glomerular sclerosis	−0.009	0.961
∆Tubular atrophy	−0.113	0.545
∆Interstitial fibrosis	−0.070	0.707
∆Fibrous crescent	0.243	0.187

r_s_: Spearman’s rho coefficient. * *p* < 0.05, ** *p* < 0.01. eGFR: estimated glomerular filtration rate, anti-dsDNA: anti-double-stranded DNA antibody, C3: complement 3, C4: complement 4, K/S ratio: kidney-to-spine ratio.

## Data Availability

Raw data are available from the corresponding author on reasonable request.
